# Molecular identification of aflatoxigenic Aspergillus species in feedstuff samples

**DOI:** 10.18502/cmm.4.2.66

**Published:** 2018-06

**Authors:** Nooshin Sohrabi, Morteza Taghizadeh

**Affiliations:** 1 Department of Biology, Payame Noor University, Tehran, Iran; 2 Department of Research and Development, Razi Vaccine and Serum Research Institute, Agricultural Research Education and Extension Organization, Karaj, Iran

**Keywords:** Aflatoxigenic, Aspergillus, HPLC, PCR, TLC

## Abstract

**Background and Purpose:**

Aflatoxins are naturally produced by some species of *Aspergillus,* such as *A.* *flavus* and* A.* *parasiticus*. Aflatoxins reportedly have carcinogenic effects on human, poultry, and livestock, and therefore could be linked to severe human illnesses. Aflatoxin biosynthesis pathway involves different clustered genes, including structural, regular, and unassigned genes. The present study was conducted to detect *aflR,* *aflP,* and* aflD* as three important genes contributing to aflatoxin B1 production cycle in *Aspergillus* species isolated from the feedstuffs of animal husbandry.

**Materials and Methods:**

This study was conducted on 25 isolates of *A. flavus*, *A. parasiticus*, *A. nomius,* and *A. nidulans,* isolated from animal feedstuff as a test group. The test group was compared with two standard strains (i.e., *A. flavus* and *A. parasiticus*) as aflatoxigenic reference organisms and negative controls (i.e., *A. fumigatus*, *A.* *fusarium,* and *A.* *penicillium*) in terms of the presence of *aflR*, *aflP*, and *aflD* genes using polymerase chain reaction (PCR). The determination of the toxigenicity and aflatoxin production of isolated *Aspergillus *species was accomplished using thin-layer chromatography (TLC) and high-performance liquid chromatography (HPLC).

**Results:**

The results obtained by the amplification of the selected genes by PCR method for the detection of aflatoxigenic *Asprgillus* species were significantly correlated with TLC and HPLC results. Accordingly, all samples, having positive results for aflatoxin B1 production in TLC and HPLC, were able to show the amplification of three target genes. However, 4 cases out of 6 (66%) non-aflatoxigenic isolates were positive for three or two genes.

**Conclusion:**

Based on the findings, the molecular detection of aflatoxin biosynthesis genes (i.e., *aflP*,* aflD,* and *aflR*) could be considered as a quick and reliable method for the detection of aflatoxigenic *Aspergillus. *Furthermore, this method could be useful in planning and implementing strategies targeted toward improving the safety of human or animal food.

## Introduction

The fungal contamination of food or feedstuff is considered as a major problem in humans or animals’ health worldwide. Nearly every food can be contaminated by fungal pathogens [1]. Many of these molds are capable of producing one or more toxins, named mycotoxins. Mycotoxins as the second metabolites of the toxigenic fungi are produced in conditions with specific humidity, temperature, and oxygen pressure [1]. 

Aflatoxins are the most important mycotoxins reported in many clinical toxicosis. These carcinogens could be accompanied with a wide range of diseases, such as cancers [2]. According to the literature, aflatoxins have teratogenic, mutagenic, and carcinogenic effects; therefore, their involvement in human food chain may be a threat to the public health [2, 3]. Furthermore, these mycotoxins have immunogenic capabilities due to having a low molecular weight. However, they can reduce resistance and interfere with induced immunity by vaccine in poultry birds [4]. 

The different species of *Aspergillus,* such as* A. flavus*,* A. parasiticus*, and* A. nomius,* are known as aflatoxin-producing fungi, which are distributed on a broad spectrum of feeds, such as corn, barley, wheat, and bran [1, 5]. Regarding this, as a great step in food safety, the contamination of human and animal food with aflatoxins should be detected with a rapid, sensitive, and highly specific method.

Previous studies have introduced molecular methods, such as polymerase chain reaction (PCR), as sensitive, specific, and powerful assays for the detection of aflatoxin [6-8]. Aflatoxin biosynthesis is a complicated pathway involving at least 18 enzymatic steps to produce these toxins from acetyl coenzyme A. Different investigations have been conducted to study the various genes involving in this process. Accordingly, they have introduced 25 genes, such as *aflP *(*omt*),* aflD *(*Nor-1*), and *aflR, *clustered in a 75-kb region of fungal chromosome III [9-11].

The *aflR* gene regulates the activity of other structural genes in the aflatoxin biosynthesis pathway. On the other hand, *aflD *(*Nor-1*) gene plays an essential role in the early conversion of norsolorinic acid into averantin. The *aflP* (*omt*) is involved in the conversion of sterigmatocystin into aflatoxin in the late steps of the aflatoxin pathway [12-14].

With this background in mind, the present study was performed to investigate the presence of *aflP*,* aflD,* and *aflR* genes in four *Aspergillus* species, isolated from animal feed samples, using the PCR method. This study also sought to evaluate the relationship of these genes with the ability of isolated fungi in aflatoxin B1 production.

## Materials and Methods

***Sampling*** ***process***

This study was conducted on a total of 25 isolates of *A. flavus*, *A. parasiticus*, *A. nomius,* and *A. nidulans,* isolated from animal feedstuff in our previous study [15]. Two standard strains, namely *A. flavus* PFCC 5004 and *A. parasiticus* PFCC 5018, were included as aflatoxigenic reference organisms. Additionally, three reference strains of non-aflatoxigenic species, including *A. fumigatus*, *A.* *fusarium,* and *A.* *penicillium*, were used as negative controls.

All of the strains were stored in distilled water (DW) and kept at room temperature for further studies. To obtain a fresh culture, the isolates were streaked in Czapek Dox agar and potato dextrose agar (Zistroyesh, Iran), allowed to grow at 25°C for 7 days.

***Extraction of fungal DNA***

The fungal DNA was extracted according to our previously described method [15]. The genomic DNA was isolated from the fungal mycelia, harvested from freshly growing cultures in potato dextrose broth. The mixture was transferred to a mortar and ground vigorously with a pestle to form a fine powder. The lysis buffer [1 M Tris-HCl (pH 7.5), 0.05 M EDTA, 0.9 M NaCl, 0.1 M Na_2_SO, and 1% sodium dodecylsulfate] was added to the mixture and subjected to a 30-min heat shock treatment at 65°C for 20 min. 

The resultant suspension was centrifuged for 5 min at 2,000 g, and the supernatant was transferred to a fresh, labled microfuge tube. The DNA extraction was performed on the supernatant using chloroform: isoamyl alcohol (24:1*) *method*.* The procedure was followed by centrifugation at 2,000 g for 15 min. The supernatant was then transferred to a fresh microcentrifuge tube, and equal volumes of isopropanol was added. Following centrifugation at 2,000 g for 5 min., the supernatant was removed, and the pellet was resuspended in 100 µl distilled water.

***Polymerase chain reaction amplification***

For the amplification of clustered pathway genes in aflatoxin biosynthesis, the PCR reaction was performed following the method described by Rahimi et al. with some modifications [16]. All of the primers were designed by OLIGO7 software, according to the reference sequence provided by the gene bank. Table 1 tabulates the specifications of primers and target genes.

All of the three genes were amplified by separate reactions following optimization. For this reason, the PCR mixture was prepared by adding 150 ng of DNA template, 1.5 mM MgCl_2_, and 10X PCR buffer containing 50 mM KCl, 1 mM dNTP, 2.5 U of taq polymerase, and 0.3 pmol of each primer, and then reached to 50 µl with distilled water. Table 2 presents the optimization of the thermal cycle of PCR reaction for each gene. The PCR products were electrophoresed on agarose gel (1%), stained with ethidium bromide, and visualized under ultraviolet light using a gel documentation system (Gel Doc 2000; Bio-Rad, Hercules, CA). 

**Table 1:** Specification and nucleotide sequence of the primers used in the study

**Gene name**	**Sequence**	**Gene length**	**Melting temperature**
*afl D (Nor-1)- F*	5′-CTCATCACACGCAGGCATCGG-3′	702	62.8
*afl D (No-r1)- R*	5′-AGATGCCTGCCACACTGTCT-3′		63.1
*afl P* (*Omt A*)-F	5′-CCCATCTCGATAGCGCCTG-3′	611	61.7
*afl P* (*Omt A*)-R	5′-GCCACCCATACCTAGATCAAAGC-3′		60.1
*afl R* – F	5′- AGAGCTACTGAACGTCCCAT-3′	1458	60.7
*afl R* – R	5′-ATCAGGTTGCACGAACTGTCC-3′		61.1

**Table 2:** Thermal cycle of polymerase chain reaction for each gene

**Gene **	**Initial denaturation**		**Denaturation** *		**Annealing** *		**Extension** *		**Final extension**	
	**Temp**	**Time**	**Temp**	**Time**	**Temp**	**Time**	**Temp**	**Time**	**Temp**	**Time**
*afl D*	95°C	3 min	95°C	30 sec	61.4°C	40 sec	72°C	30 sec	72°C	7 min
*afl P*	95°C	3 min	95°C	30 sec	60°C	45 sec	72°C	45 sec	72°C	7 min
*afl R*	95°C	2 min	95°C	30 sec	58°C	45 sec	72°C	90 sec	72°C	7 min

* The denaturation, annealing, and extension were repeated for 34 cycles for each gene.

***Determination of aflatoxin production***

***a) Fluorescence observation on plate***

The ability of all fungal samples for aflatoxin production was detected on coconut agar medium (CAM). To this end, the fungi were cultured on CAM and incubated at 30°C in the dark for 7-10 days. The plates were periodically screened for blue fluorescence under ultraviolet (UV) light (365 nm).

***b) Qualitative determination of aflatoxin by*** ***thin-layer chromatography***

The assessment of aflatoxin levels in isolates was performed after initial extraction. To this end, the samples were cultured in 100 ml yeast extract-sucrose broth medium (Merck, Germany) and incubated in the dark for 7 days at 30°C. Afterwards, the culture was finely mixed with NaCl and methanol 55% for 30 min and filtered through Whatman filter paper No. 1. Then, 100 ml chloroform was added to culture flask and centrifuged at 1,500 g for 15 min at 4°C. Subsequently, the lower phase was transferred to a new tube and evaporated at 80°C for the removal of chloroform. 

For the evaluation of aflatoxin-producing ability of fungal isolates, 20 µl of prepared extract was transferred to silica gel paper by Dispenser Capillary for TLC. After the development of plates by chloroform:acetone (1:9) protocol, the fluorescence intensities of the samples were observed under UV light. 

***c) Quantitative determination of aflatoxin B1 by high-performance liquid chromatography***

The concentrations of aflatoxin B1 were determined according to a method described by Namjoo [17] using high-performance liquid chromatography (HPLC) with a fluorescent detector (Knaur, Germany). To this end, 20 µl of toxin extract was injected into the HPLC reversed-phase columns C18 column (TSKgel ODS- 210,4.6 mm ID×150 mm, Tosoh Bioscience, Japan) and washed at a flow rate of 1 ml/min by water: methanol: acetonitrile (60:30:15 v/v/v). The fluorescence was detected at the excitation and emission wavelengths of 365 and 440 nm, respectively. 

The B1 aflatoxin standards were provided from Sigma (St. Louis, MO). Additionally, the different concentrations of aflatoxin B1 standards (i.e., 5, 10, 100, 500, and 1000 ng/ml) were analyzed by HPLC, according to which a standard curve was prepared. The quantities of aflatoxin B1 in samples were detected by comparing the under the curve area of the chromatogram with the standard curve. The total recovery was obtained as 93.4%.

## Results

In this study, four *Aspergillus *species*,* including *A. flavus* (n=17),* A. parasiticus *(n=5), *A. nomius* (n=1), and *A. nidulans* (n=2), were isolated from the feedstuff samples. All of the species were identified by their macroscopic and microscopic features and sequencing of the internal transcribed spacer region. The presence of *aflP*,* aflD,* and *aflR* genes were evaluated by PCR using three sets of primers. 

Figure 1a-c depicts the PCR products obtained from each gene fragments. The separate bands of *aflD*,* aflP,* and *aflR* gene fragments could be seen at 702, 611, and 1458 bp, respectively. A heterogenic pattern was observed in the positive and negative controls. A similar electrophoretic pattern was observed on the PCR products of 20 isolates, which represented the concomitant presence of the three genes. However, other isolates demonstrated different patterns. 

**Figurer 1:**
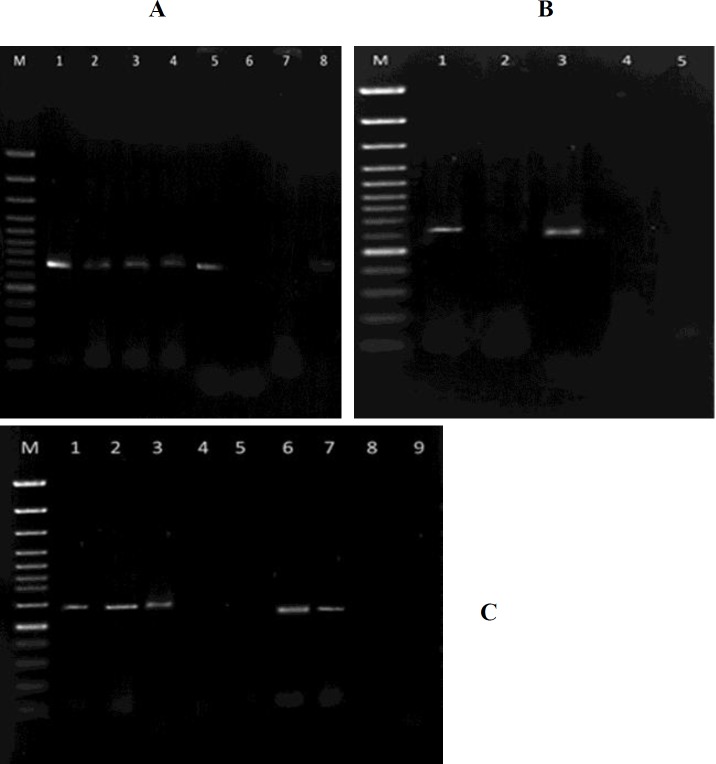
Detection of *aflR*, *aflP,* and* aflD* genes in genomic DNA extracted from *Aspergillus* species by polymerase chain reaction (PCR) using a separated primer set. The amplified PCR products were analyzed by 1% agarose gel electrophoresis. The specific band corresponding to expected molecular size of *aflD* (A) (702 bp, Lane 1-5), *aflP* (B) (611 bp, lane 1 and 3), and *aflR* (C) (1458 bp, lane 1-3 and 6, 7) were detected. Lane M indicates the 100 bp (A and B) and 1 Kb (C) DNA ladder size marker.

**Table 3:** Comparision of the results of conventional and molecular assays in terms of aflatoxin production

**Number**	***Aspergillus *** **species**	**Source**	**Aflatoxin B1 (HPLC, ppb)**	**CAM** **fluorescence**	**Aflatoxin production on TLC**	**Detection by PCR**		
						***afl P***	***afl D***	***afl R***
1	*A. flavus*	Imported barley	21.85	+	Positive	+	+	+
2	*A. flavus*	Imported barley	0.05	-	Positive	+	+	+
3	*A. flavus*	Soybean meal	ND	-	Negative	+	+	+
4	*A. flavus*	Soybean meal	0.41	+	Positive	+	+	+
5	*A. flavus*	Imported barley	45.01	+	Positive	+	+	+
6	*A .flavus*	Soybean meal	0.13	+	Positive	+	+	+
7	*A. flavus*	Iranian barley	ND	+	Negative	-	-	-
8	*A. flavus*	Iranian barley	6.1	+	Positive	+	+	+
9	*A. flavus*	Soybean meal	0.16	+	Positive	+	+	+
10	*A. flavus*	Soybean meal	11.58	+	Positive	+	+	+
11	*A. flavus*	Corn	ND	-	Negative	-	+	+
12	*A. flavus*	Corn	21.43	+	Positive	+	+	+
13	*A. flavus*	Imported barley	16.8	+	Positive	+	+	+
14	*A. flavus*	Bran	ND	-	Negative	-	-	-
15	*A. flavus*	Bran	23.1	+	Positive	+	+	+
16	*A. flavus*	Imported barley	37.2	+	Positive	+	+	+
17	*A. flavus*	Corn	0.83	+	Positive	+	+	+
18	*A. parasiticus*	Iranian barley	ND	-	Negative	+	-	+
19	*A. parasiticus*	Soybean meal	12.36	+	Positive	+	+	+
20	*A. parasiticus*	Corn	25.48	+	Positive	+	+	+
21	*A. parasiticus*	Iranian barley	0.62	+	Positive	+	+	+
22	*A. parasiticus*	Imported barley	ND	-	Negative	+	+	+
23	*A. nomius*	Imported barley	8.11	+	Positive	+	+	+
24	*A. nidulans*	Corn	14.8	+	Positive	+	+	+
25	*A. nidulans*	Soybean meal	35.21	+	Positive	+	+	+
26	*A. parasiticus (PFCC 5018)*	Standard strain	707.9	+	Positive	+	+	+
27	*A. flavus *(PFCC 5004*)*	Standard strain	108.8	+	Positive	+	+	+

*A. flavus *PFCC 5004 and *A. parasiticus* PFCC 5018

HPLC: high-performance liquid chromatography, CAM: coconut agar medium, TLC: thin-layer chromatography, PCR: polymerase chain reaction

As shown in Table 3, the responsible aflatoxin biosynthesis genes (i.e., *aflD*,* aflP,* and *aflR*), which were greatly detected by PCR method in the positive aflatoxigenic samples, were absent in the negative control species.

The TLC and HPLC methods and the PCR amplification rendered completely similar results regarding the prevalence of target genes and aflatoxin B1 production ability (Table 3). As seen in Table 3, all of fungal isolates that were positive for aflatoxin production based on the TLC and HPLC assays could amplify *aflD*,* aflP,* and *aflR* genes. On the other hand, 4 cases out of 25 samples (16%), which were detected as aflatoxin non-producing *Aspergillus*, showed a complete set or both of genes. Only 2 (8%) samples, which were negative for aflatoxin production, had negative results in PCR and other conventional methods.

## Discussion

Given the technical difficulties of conventional methods in the detection of aflatoxin-producing *Aspergillus*, scientists have opted for new and sensitive methods for the early detection of foodstuff contamination with different aflatoxigenic* Aspergillus* species [18-20]. Among the different genes involving in aflatoxin biosynthesis pathway, *aflD* plays an important role in the early conversion of nosolorinic acid into averantin. On the other hand, *afl P* is involved in the conversion of strigmatocystin into aflatoxin in the late steps. Moreover, *afl R *gene has a key role in the regulation of other genes in aflatoxin biosynthetic pathway [21-23].

In the present study, 92% (n=23) of aflatoxigenic *Aspergillus* species gave consistent results regarding aflatoxin production on CAM as confirmed by TLC and HPLC. However, there was a complete correlation between the results of these tests about the aflatoxin-producing isolates by the presence of three candidate genes and those of other conventional methods. However, 8% (n=2) of the test samples, which were considered as non-producing strains, had negative responses in fluorescence, TLC, and HPLC methods, and showed no bands in PCR reaction for each separated gene. This finding is consistent with those of the previous studies [19, 20].

Previously, we investigated the effect of aflatoxin genes, namely *aflP* and *aflQ,* on aflatoxigenic species of *A. flavus *and* A. parasiticus* in cattle feed. In the mentioned study, 67.16% and 70.14% of the samples had *aflP* and *aflQ, *respectively, and the HPLC findings also confirmed this result [15].

Degola et al. developed a multiplex real time-PCR protocol based on *aflD*, *aflO,* and *aflQ* genes for the discrimination of aflatoxin-producing strains of *A. flavus* from the aflatoxin-nonproducing ones. They reported a good correlation between the target genes expression in nearly all samples [24]. In another study, an optimized multiplex PCR was developed based on *avfA, omtA, *and* ver-1 *genes for the rapid and sensitive detection of potential aflatoxigenic molds in fermented foods and grains [25].

Houssain *et al.* reported that the presence of *Aspergillus *structural genes (i.e., *Nor-1 *and* Ver-1*) and regulatory gene (i.e., *aflR*) could be considered as an early indicator of aflatoxin production [26]. In the current study, we found two isolates, which were positive for *aflR* and negative for *aflD* or *aflP* genes. It was observed that all of* A. flavus* and *A. parasiticus* samples could amplify *aflR* gene. Nonetheless, the presence of this gene is not sufficient for aflatoxin production. These results showed that many other genes and factors account for the aflatoxigenicity of organisms [27]. Schmidt showed that environmental factors have a complex influence on the regulation of aflatoxin biosynthesis genes [28].

Inconsistent with previous reports [19], some non-aflatoxigenic strains of *A. flavus* and *A. parasiticus,* which showed negative results in the conventional methods, could express at least one of the *aflP*, *aflD,* or *aflR* genes. In line with the previous studies, our results indicated that the lack of aflatoxin production in some aflatoxigenic species could be due to the deletion and simple mutation (replacement of some bases) of *aflR*, *aflD,* and *aflP* genes or loss of other responsible genes in aflatoxin production pathway. However, some of the physiological conditions could affect aflatoxin biosynthesis [12, 21].

Furthermore, the additional molecular character-rization of these genes or other eventual genes in aflatoxin biosynthesis pathway could be useful for the application of these methods in differentiating between toxinogenic and nontoxinogenic strains of *Aspergillus*. The results of this study could be helpful for agricultural development and medical or veterinary organization in aflatoxin regulatory controls.

## Conclusion

As the findings of the present study indicated, the molecular detection of aflatoxin biosynthesis genes (i.e., *aflP*,* aflD,* and *aflR*) could be considered as a quick and reliable method for the identification of aflatoxigenic *Aspergillus *species. Moreover, this method could be useful in planning and implementing the strategies targeted toward improving the safety of human or animal food.
